# A defined method for differentiating human iPSCs into midbrain dopaminergic progenitors that safely restore motor deficits in Parkinson’s disease

**DOI:** 10.3389/fnins.2023.1202027

**Published:** 2023-07-12

**Authors:** Ryota Nakamura, Risa Nonaka, Genko Oyama, Takayuki Jo, Hikaru Kamo, Maierdanjiang Nuermaimaiti, Wado Akamatsu, Kei-ichi Ishikawa, Nobutaka Hattori

**Affiliations:** ^1^Department of Neurology, Faculty of Medicine, Juntendo University, Tokyo, Japan; ^2^Department of Diagnosis, Prevention and Treatment of Dementia, Graduate School of Medicine, Juntendo University, Tokyo, Japan; ^3^Department of Clinical Data of Parkinson’s Disease, Graduate School of Medicine, Juntendo University, Tokyo, Japan; ^4^Center for Genomic and Regenerative Medicine, Graduate School of Medicine, Juntendo University, Tokyo, Japan; ^5^Department of Research and Development for Organoids, School of Medicine, Juntendo University, Tokyo, Japan; ^6^Neurodegenerative Disorders Collaborative Laboratory, RIKEN Center for Brain Science, Saitama, Japan

**Keywords:** Parkinson’s disease, iPS cells, regenerative medicine, cell transplantation, dopaminergic neurons

## Abstract

**Background:**

Parkinson’s disease (PD) is a progressive neurodegenerative condition that primarily affects motor functions; it is caused by the loss of midbrain dopaminergic (mDA) neurons. The therapeutic effects of transplanting human-induced pluripotent stem cell (iPSC)-derived mDA neural progenitor cells in animal PD models are known and are being evaluated in an ongoing clinical trial. However, However, improvements in the safety and efficiency of differentiation-inducing methods are crucial for providing a larger scale of cell therapy studies. This study aimed to investigate the usefulness of dopaminergic progenitor cells derived from human iPSCs by our previously reported method, which promotes differentiation and neuronal maturation by treating iPSCs with three inhibitors at the start of induction.

**Methods:**

Healthy subject-derived iPS cells were induced into mDA progenitor cells by the CTraS-mediated method we previously reported, and their proprieties and dopaminergic differentiation efficiency were examined *in vitro*. Then, the induced mDA progenitors were transplanted into 6-hydroxydopamine-lesioned PD model mice, and their efficacy in improving motor function, cell viability, and differentiation ability in vivo was evaluated for 16 weeks.

**Results:**

Approximately ≥80% of cells induced by this method without sorting expressed mDA progenitor markers and differentiated primarily into A9 dopaminergic neurons in vitro. After transplantation in 6-hydroxydopamine-lesioned PD model mice, more than 90% of the engrafted cells differentiated into the lineage of mDA neurons, and approximately 15% developed into mature mDA neurons without tumour formation. The grafted PD model mice also demonstrated significantly improved motor functions.

**Conclusion:**

This study suggests that the differentiation protocol for the preparation of mDA progenitors is a promising option for cell therapy in patients with PD.

## Introduction

Parkinson’s disease (PD) is the second most common neurodegenerative disorder. The prevalence of PD is approximately 1% in people aged above 60 years; it progresses with age, and will subsequently result in a future increase in the number of patients affected (de Lau and Breteler, 2006; Kalia and Lang, 2015). PD primarily affects the patient’s motor functions due to the progressive loss of midbrain dopaminergic (mDA) neurons projected to the striatum (Kalia and Lang, 2015). Currently, palliative treatment includes dopamine replacement medications and neuromodulation therapies (such as deep brain stimulation) (Jakobs et al., 2019). However, the development of disease-modifying therapies is essential to address the worsening of symptoms due to disease progression. Cell replacement therapy, involving the transplantation of dopaminergic cells in the striatum, is a potential strategy for disease modification (Barker et al., 2015, 2017; Parmar et al., 2019; Schweitzer et al., 2020).

The first projected cellular resource of mDA neurons was the foetal ventral midbrain (fVM) dopaminergic cells. After obtaining positive results in PD-model animals (Brundin et al., 1986), the first clinical trial of fVM cell transplantation to patients with PD was performed in Sweden in 1987 (Lindvall et al., 1989), and the results of many subsequent open-labeled studies have generally been favorable (Piccini et al., 1999; Kefalopoulou et al., 2014). However, two double-blind studies conducted in the 1990s did not show statistically significant improvement and revealed that the transplantation of fVM cells causes graft-induced dyskinesia (GID) in some patients (Freed et al., 2001; Olanow et al., 2003). In addition, fVM transplantation has drawbacks such as limited tissue supply and ethical issues. Therefore, fVM cells could not be used in standard transplantation therapy. In contrast, a good long-term prognosis has been reported in the post-transplant brain pathology of patients after fVM transplantation and engraftment of transplanted cells (Kefalopoulou et al., 2014). Subsequent analysis revealed that the effect of transplantation is associated with the disease stage, patient’s age, number of grafted cells, and the duration of immunosuppression treatment (Freed et al., 2001; Barker et al., 2013; Lindvall, 2013). Among several factors considered to determine the cause of GID, the contamination of serotonergic neurons may be the primary cause (Barker and Kuan, 2010; Politis et al., 2010; Steece-Collier et al., 2012). Therefore, if these issues are addressed, cell therapy could be a valuable approach to controlling PD progression in limited cases.

Human embryonic stem cells (ESCs) introduced in 1998 (Thomson et al., 1998), and human induced pluripotent stem cells (iPSCs) introduced in 2007 (Takahashi et al., 2007), are potent resources for regenerative medicine. In particular, iPSC-derived cells are ethically accepted, and their autografting is theoretically unaffected by immune mechanisms. For the practical use of iPSCs, establishing a robust differentiation method to induce highly enriched mDA neuron progenitors is critical for efficient treatment, and avoiding tumourigenesis or other adverse events. Owing to several basic experiments, methods for the induction of functional mDA neurons via floor plate cells have improved (Chambers et al., 2009; Fasano et al., 2010; Kriks et al., 2011; Kirkeby et al., 2012; Nolbrant et al., 2017). It is based on the combination of dual SMAD inhibition for neural lineage induction with Wnt activation for defining the midbrain patterning and ventralisation through an SHH agonist (Kim et al., 2021). More recently, human iPSC-derived cells sorted with cell surface mDA progenitor markers such as ALCAM (Bye et al., 2015), CORIN (Doi et al., 2014; Kikuchi et al., 2017; Doi et al., 2020), and LRTM1 (Samata et al., 2016) have proven to be safe and effective when transplanted into animal PD models. In 2018, Takahashi and colleagues initiated a clinical trial of cell transplantation therapy for patients with PD to investigate the safety and efficacy of iPSC-derived CORIN-positive mDA progenitors (Takahashi, 2020). These cells are differentiated from allogenic iPSCs, because autotransplantation is difficult due to the cost of generating safe, clinical-grade iPSC lines. To provide cell therapy to a large population of patients, it is necessary to prepare a large number of cells as therapeutic products. The induction method currently used in clinical trials sorts 18.9% or 31.4% CORIN-positive cells (Doi et al., 2014, 2020); therefore, improvements in the safety and efficiency of differentiation inducing methods are crucial for providing a larger scale of cell therapy studies. In line with this concept, Kim et al. (2021) and Piao et al. (2021) reported expandable mDA progenitor cells derived from embryonic stem cells for transplantation into patients with PD by improving their induction with adhesion culture, called the ‘floor plate (FP)-method’ (Kim et al., 2021; Piao et al., 2021), and a clinical trial has been started in the US.

Recently, our research group reported a method to promote differentiation efficiency and maturation of iPSC-derived cells by treatment with three small molecules, SB431542 (SB, a SMAD signal inhibitor), dorsomorphin (DM, a bone morphogenetic protein signal inhibitor), and CHIR99021 (CHIR, a GSK3 inhibitor) (Fujimori et al., 2017). Treatment of iPSCs with these inhibitors enhances their differentiation into three germ layers (named chemically transitional embryoid-body-like state; CTraS) and accelerates their differentiation into neurons following neural induction. Tyrosine hydroxylase (TH, a dopaminergic neuron marker)-positive mDA neurons are also efficiently induced by this method following induction into the ventral midbrain neuron. Moreover, it is possible to replicate *in vitro* disease-specific pathological phenotypes using multiple iPSCs derived from patients with hereditary PD (Shiba-Fukushima et al., 2017; Suzuki et al., 2017; Ikeda et al., 2019; Oji et al., 2020; Yamaguchi et al., 2020). We therefore hypothesized that the mDA progenitors developed by this method have adequate efficacy and safety for the cell therapy of patients with PD.

This study aimed to propose a novel method based on CTraS-mediated induction, to prepare iPSC-derived mDA progenitors for cell transplantation therapy in patients with PD. The transplanted mDA progenitors induced by our protocol without sorting were successfully integrated in the striatum of a 6-hydroxydopamine (6-OHDA)-induced PD mice model and improved motor symptoms without tumor formation. With the advantages of ease of applicability and scalability to future large-scale culture systems, this protocol is a promising alternative for cell therapy.

## Materials and methods

### Human iPSC culture

The human iPSC line, 201B7 (Takahashi et al., 2007), was obtained from Kyoto University via RIKEN BioResource Research Centre in accordance with the relevant guidelines and regulations. The iPSCs were cultured on mitomycin C-treated SNL murine fibroblast feeder cells in human iPSC medium, according to a previous report (Takahashi et al., 2007). All experimental procedures were approved by the Juntendo University School of Medicine Ethics Committee and all experiments with human iPSCs were performed in accordance with relevant guidelines and regulations.

### *In vitro* neuronal induction

Differentiation into mDA neurons from human iPSCs was performed as described previously (Imaizumi et al., 2015; Matsumoto et al., 2016; Fujimori et al., 2017; Ishikawa et al., 2018; Yamaguchi et al., 2020) with minor modifications. The iPSCs cultured on feeder cells were treated with 3 μM aSB431542 (Tocris Bioscience, Avonmouth, UK), 3 μM dorsomorphin (Sigma-Aldrich, St. Louis, MO, USA), and 3 μM CHIR99021 (REPLOCELL, Yokohama, Japan) on day 0 in the human iPS medium. The medium was replenished with the three chemicals every day for 5 days for differentiation into the three germ layers with a chemically transitional EB-like state (CTraS) (Fujimori et al., 2017). To generate non-CTraS-mediated mDA progenitors for evaluating tumorigenesis, iPSCs were cultured for 5 days in the absence of these three small molecules. On day 5, the iPSC colonies were detached from the feeder layers using a dissociation solution (REPLOCELL) and enzymatically dissociated into single cells using TrypLE Select (Life Technologies, Carlsbad, CA, USA) at 37°C for 5–7 min. The dissociated and filtered (40 μm) cells were cultured in a suspension at a density of 1 × 10^4^ cells/mL in the neurosphere medium in 4% O_2_, to form primary neurospheres. The neurosphere medium is a KBM Neural Stem Cell medium (KOHJIN BIO, Saitama, Japan) supplemented with 1% penicillin–streptomycin (Life Technologies), 2% B27 supplement (Life Technologies), 20 ng/mL basic fibroblast growth factor (bFGF; Pepro Tech, Rocky Hill, NJ, USA), 2 μM SB431542 (Tocris Bioscience), and 5 μM Y27632 (Wako, Osaka, Japan). For ventral midbrain specification, 3 μM CHIR99021 and 2 μM purmorphamine (Millipore, Burlington, MA, USA) were added to the culture medium on day 8. For forebrain specification, we added 3 μM IWR-1-endo (Millipore) on day 5, and 2 μM purmorphamine on day 8. For spinal cord specification, we added 3 μM CHIR and 1 μM retinoic acid (Sigma-Aldrich, St. Louis, MO, USA) on day 5 and 2 μM purmorphamine on day 8. On day 19, the corrected neurospheres were centrifuged for 5 min at 200× *g* and the supernatant was separated. Further, the neurospheres were dissociated using TrypLE Select and filtered using the same procedures as performed on day 5. Some dissociated neurospheres on day 19 were passaged in suspension at a density of 5 × 10^4^ cells/mL in the neurosphere medium with 3 μM CHIR99021 and 2 μM purmorphamine, and cultured for another 7–10 days, to form secondary neurospheres. For the *in vivo* transplantation experiments, dissociated neurospheres were used. For *in vitro* neural differentiation, the dissociated neurospheres were plated onto 6-well plates or 8-well chamber slides coated with poly-L-ornithine (Sigma-Aldrich) and fibronectin (Corning, NY, USA). These cells were cultured in the neuron medium consisting of KBM Neural Stem Cell medium supplemented with 2% B27 supplement, 20 ng/mL brain-derived neurotrophic factor, (BDNF) (BioLegend, San Diego, CA, USA), glial cell-derived neurotrophic factor (GDNF) (PEPROTECH Inc., Rocky Hill, NJ,USA), 200 μM ascorbic acid (Sigma-Aldrich, St. Louis, MO, USA), 0.5 mM dibutyryl-cyclic adenosine monophosphate (Nakalai Tesque, Kyoto, Japan), 1 ng/mL transforming growth factor β3 (TGF-β3; BioLegend), and 10 μM DAPT (Sigma-Aldrich). Cells were cultured for 17 days in a humidified atmosphere containing 5% CO_2_. CHIR (3 μM) was added to this medium only on day 19. Half of the medium was changed every 2 days.

### Quantitative real-time PCR

Total RNA was isolated using the RNeasy mini kit (QIAGEN, Hilden, Germany) with DNase I treatment, and cDNA was generated using a ReverTraAce qPCR RT kit (TOYOBO, Osaka, Japan). The qPCR analysis was performed with SYBR premix Ex Taq II (Takara Bio, Shiga, Japan) on a ViiA™ 7 real-time PCR system (Thermo Fisher Scientific, Waltham, MA, USA). Values were normalized to *ACTB* and analyzed using the comparative (ΔΔCt) method. Primers used in this study are listed in Supplementary Table S1.

### Transplantation into the mice PD models

Male CB17-SCID mice (8-week-old) were purchased from Charles River Laboratories (Yokohama, Japan). Animals were cared for in accordance with the Guiding Principles for the Care and Use of Animals approved by the Ethics Review Committee for Animal Experimentation of Juntendo University School of Medicine, and we performed all mouse experiments in accordance with the approved protocol. All experiments were carried out in compliance with the ARRIVE guideline and the relevant guidelines and regulations. The mice were stereotaxically injected 6-OHDA (Sigma-Aldrich) into the right striatum to generate a Parkinsonian model (Lundblad et al., 2004). Each mouse received two unilateral injections of 6 μg 6-OHDA in 2 μL of saline with 0.02% ascorbic acid.

The coordinates were calculated with reference to bregma (in mm from bregma): AP +1, ML −2.1, DV −3.2, and AP +0.3, ML −2.3, DV −3.2 (Bateup et al., 2010). Three weeks after the 6-OHDA injection, the transplantation group received cell transplantation via a stereotactic injection of 2 × 10^5^ dissociated neurospheres in 2 μL KBM Neural Stem Cell medium through a 22G needle into the right striatum at the following coordinates (in mm from bregma): AP +0.5, ML +2.0, DV −3.8 (Steinbeck et al., 2015). Eleven (of 18) model mice were transplanted with primary neurospheres and seven were transplanted with secondary neurospheres. The sham group received injections of the same amount of saline into the same coordinates. Sixteen weeks after transplantation, the animals were euthanised with pentobarbital and perfused transcardially with 10% formalin (Wako). The brains were removed immediately and saturated with phosphate-buffered saline (PBS) containing 30% sucrose. The brains were cut using a cryostat (CM-1850; Leica Biosystems) at 30 μm thickness and mounted for the immunofluorescence study.

### Behavior analysis

The apomorphine-induced rotational behavior assay was performed 1 week before and every 4 weeks after transplantation. A dose of 0.5 mg/kg of apomorphine (Wako) was injected subcutaneously, and the rotations were recorded for 30 min (Akerud et al., 2001; Han et al., 2015; Zuo et al., 2017). The animal behavior was automatically calculated using DAQ PL3508 PowerLab 8/35 ADInstruments Australia (Software LabChart ver.8 ADInstruments Australia). Only mice that rotated seven or more rotations per minute 2 weeks after 6-OHDA injection were defined as PD model mice.

### Immunofluorescence studies

For *in vitro* studies, cells were fixed with 4% paraformaldehyde for 15 min at 20–24°C. The fixed cells were blocked with 5% normal foetal bovine serum and 0.3% Triton X-100 for 1 h at 20–24°C, and incubated with primary antibodies diluted with blocking solution overnight (15–20 h) at 4°C. After reaction with the primary antibodies, the samples were washed with PBS and incubated with species-specific secondary antibodies conjugated with Alexa Fluor 488, 594, and 647 (1:500, Thermo Fisher Scientific) for 1 h at 20–24°C. These samples were mounted on slides with mounting medium containing DAPI (Vector Laboratories Inc., Burlingame, CA, USA). For *in vivo* studies, brain sections were blocked with 1% normal horse serum and 0.05% Triton X for 1 h at 20–24°C and then incubated with primary antibodies diluted with blocking solution overnight (15–20 h) at 4°C using the free-floating method. The samples were washed with PBS and incubated with species-specific secondary antibodies conjugated with Alexa Fluor 488, 594, and 647 (1,500) for 1 h at 20–24°C. These samples were mounted on slides with mounting medium containing DAPI (Vector Laboratories Inc.). These images were examined using laser scanning confocal microscopy (TCS-SP5; Leica Biosystems), an LSM-710 confocal laser-scanning microscope (Carl Zeiss, Jena, Germany) and a BZ-9000 fluorescence microscope (Keyence, Osaka, Japan). The *in vitro* positivity rate was calculated using ImageJ/Fiji (version 1.53c). The cell positive rate in the brain graft was calculated from the average of 1–3 different fields at a magnification of 40× using sections in which the graft remains as large as possible. Antibodies used in this study are listed in Supplementary Table S2.

### Statistical analyses

Statistical significance between the two samples was determined using the Student’s t-test (SPSS Version. 25.0; SPSS, Inc., Chicago, IL, USA). The data were considered statistically significant at *p* < 0.05 and are shown as mean ± standard error of the mean.

## Results

### The human iPSC-derived mDA progenitors induced using the neurosphere-based protocol have ventral midbrain identity and efficiently differentiate into mDA neurons

**Figure 1:**
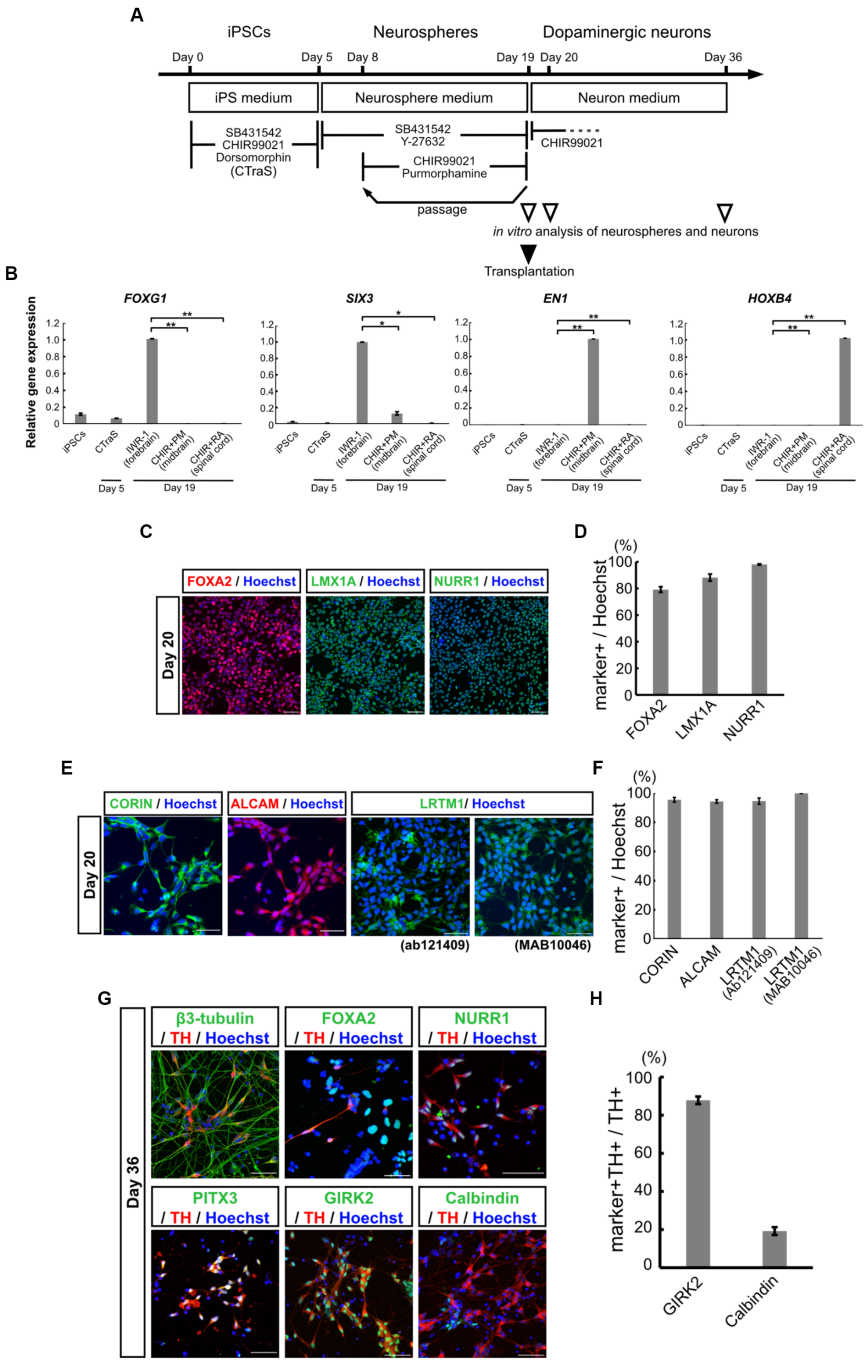
Characterization of mDA progenitors and neurons induced using the neurosphere-based differentiation protocol. **(A)** Overview of the differentiation protocol using chemically transitional embryoid-body-like state (CTraS) method from human iPSCs to dopaminergic neurons. The treatment with three chemicals of iPSCs induces CTraS. **(B)** qRT-PCR analysis of anteroposterior markers in neurospheres derived from iPSCs (All samples *n* = 3, mean ± standard error of mean [SEM]). Values were normalized to *ACTB* and were analyzed using the comparative (ΔΔCt) method. *p < 0.05, **p < 0.01. **(C–F)** Immunostaining analysis of mDA progenitors on day 20. Immunofluorescence images of mDA progenitor markers (FOXA2, LMX1A, NURR1, CORIN, ALCAM, and LRTM1) **(C,E)** and their positivity rates **(D,F)**. **(G,H)** Immunostaining analysis of the differentiated mDA neurons *in vitro* on day 36. Immunofluorescence images of the mDA neuron markers **(G)** and the positive rate of GIRK2, an A9 mDA neuron marker, and Calbindin, an A10 mDA neuron marker for TH-positive cells **(H)**. Bars, 50 μm. mDA, midbrain dopaminergic; qRT-PCR, quantitative real-time Polymerase Chain Reaction; iPSCs, induced pluripotent stem cells; N.D., not detected.

The iPSCs derived from a healthy volunteer were differentiated into mDA progenitors, according to our previous reports (Imaizumi et al., 2015; Matsumoto et al., 2016; Fujimori et al., 2017; Yamaguchi et al., 2020) as summarized in Figure 1A. Human iPSCs were treated with SB, DM, and CHIR for 5 days to develop the CTraS condition, which expresses the genes specific to the three germ layers and the embryoid bodies *in vitro*. The dissociated cells were then transferred to a floating culture to form neurospheres from each single neural stem cell (Matsumoto et al., 2016; Fujimori et al., 2017). These neurospheres were formed with purmorphamine (a sonic hedgehog agonist) and CHIR to provide the ventral midbrain regional identity as mDA progenitors (Imaizumi et al., 2015; Yamaguchi et al., 2020). To verify the ventral midbrain regional identity of the neurospheres, we performed qPCR analysis using region-specific markers on day 19. We used neurospheres treated with IWR-1-endo, a Wnt antagonist that provides a forebrain specification; forebrain spheres and neurospheres treated with RA provide posterior hindbrain and spinal cord specification, as spinal-cord spheres. FOXG1, a frontal cortex marker, and SIX3, a forebrain marker, were expressed in IWR-1-endo-treated forebrain neurospheres, but not in purmorphamine and CHIR-treated midbrain neurospheres. EN1, a floor plate midbrain marker, was expressed only in midbrain neurospheres. HOXB4, a hindbrain marker, was expressed in CHIR and RA-treated spinal cord neurospheres, but not in the midbrain or forebrain neurospheres (Figure 1B). These results indicate that neurospheres differentiated from iPSCs acquired ventral midbrain regional identity using purmorphamine and CHIR.

On day 19, the midbrain neurospheres were dissociated and plated onto culture dishes for terminal differentiation into dopaminergic neurons. Immunostaining of neural progenitor cells after one day in adhesive culture (day 20) confirmed that the cells were highly positive for mDA progenitor markers, FOXA2, LMX1A, and NURR1 (Figure 1C). The positivity rates for these markers in all the cells were 79.2 ± 2.11%, 88.2 ± 2.63%, and 98.0 ± 0.79%, respectively (Figure 1D). We also confirmed the expression of EN1 and these markers using qPCR (Supplementary Figures S1A–D). Additionally, 95.4 ± 1.65% of the progenitor cells were positive for the floor plate surface marker CORIN (Figures 1E,F), which has been used for sorting to enrich midbrain progenitors in the ongoing clinical trial by Takahashi and colleagues (Doi et al., 2014; Kikuchi et al., 2017; Doi et al., 2020; Takahashi, 2020). ALCAM (a central nervous system microvascular endothelium marker), and LRTM1 (a ventral midbrain marker), which have been reported as mDA progenitor markers for transplant therapy (Bye et al., 2015; Samata et al., 2016), were positive in most of the progenitors; 94.2 ± 1.56% positive for ALCAM, 94.5 ± 2.13% positive for an LRTM1 antibody from Abcam (Ab121409) and 99.8 ± 0.11% positive for another LRTM1 antibody from R and D (MAB10046) (Figures 1E,F). The expression of these mDA markers was also validated using qPCR (Supplementary Figures S1E–G). Recently, Xu et al. (2022) proposed other mDA progenitor markers, CLSTN2 and PTPRO, and successfully enriched mDA neurons by sorting, using CLSTN2-tdTomato and PTPRO-tdTomato knock-in hiPSCs due to the lack of commercially available antibodies (Xu et al., 2022). We also confirmed the expression of CLSTN2 and PTPRO using qPCR (Supplementary Figures S1H,IH,I). As expected, these results indicate that midbrain neurospheres differentiating from iPSCs contained mDA neural progenitors.

To determine the ability of mDA progenitors to differentiate into mDA neurons *in vitro*, the differentiated neurons were evaluated using immunostaining and qPCR analysis on day 36 (Figure 1G; Supplementary Figure S1J–O). The cells were differentiated into neurons (β3-tubulin+, a neuron marker/nucleus, 81.9 ± 1.27%) and enriched with mDA neurons (TH+/β3-tubulin+, 61.0 ± 9.23%). Most of the TH+ cells also expressed mDA markers such as NURR1 and PITX3, and mature mesencephalic dopaminergic neuron markers (Figure 1G). Among the TH-positive neurons, 87.8 ± 1.95% were positive for GIRK2, an A9 mDA neuron marker, and 19.3 ± 2.05% were positive for Calbindin, an A10 mDA neuron marker (Figures 1G,H). These results indicate that the neurosphere-based mDA neuron induction method efficiently induced ventral mDA progenitors from iPSCs without fluorescence-activated cell sorting (FACS), and the progenitors differentiated into mature mDA neurons, which were predominantly A9 neurons *in vitro*.

### Human iPSC-derived mDA progenitors transplanted into PD model mice successfully differentiated into mDA neurons *in vivo*

**Figure 2:**
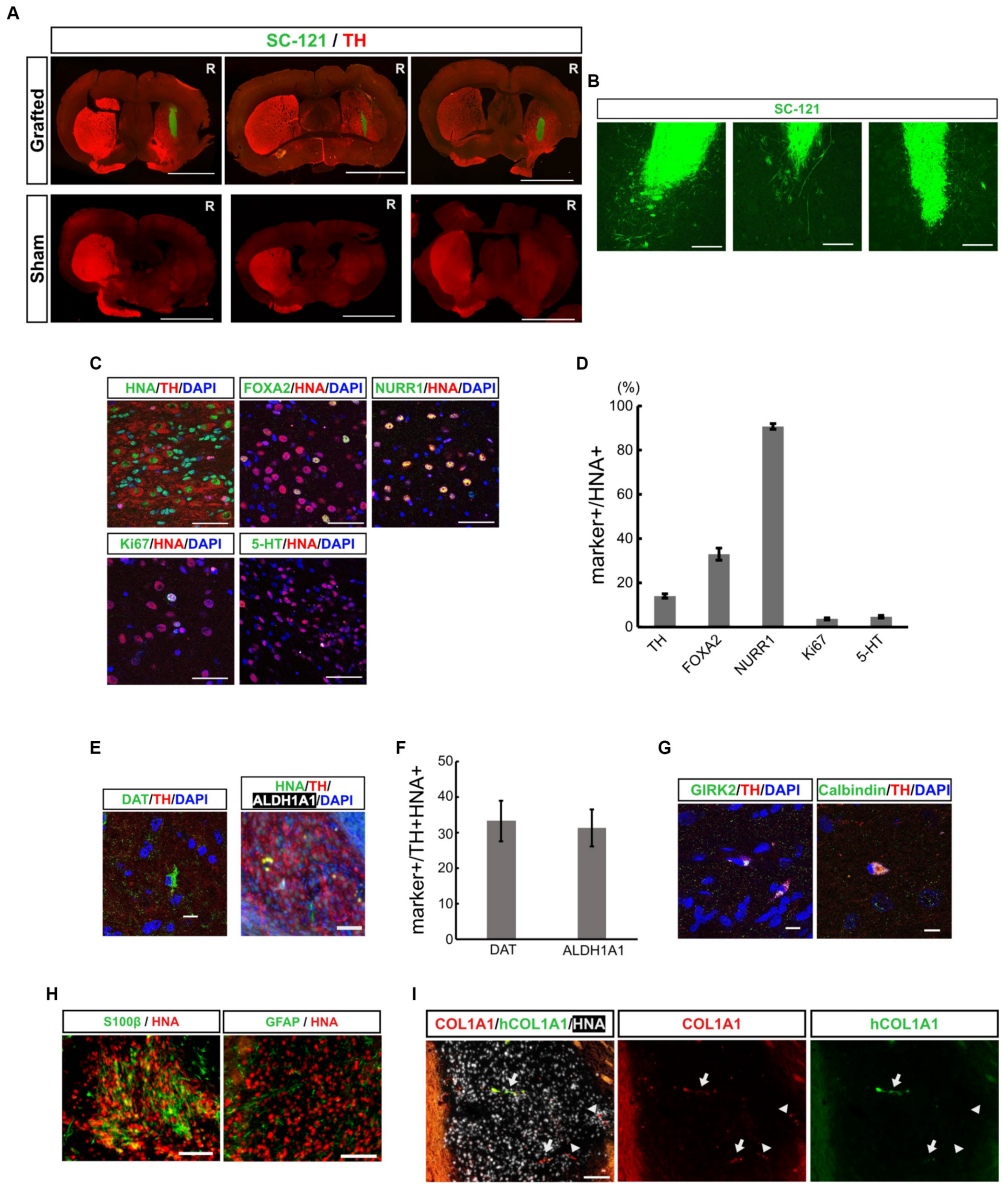
Grafted mDA progenitors using a neurosphere-based protocol differentiate into mDA neurons in the brains of 6-OHDA-induced PD-model mice. **(A)** Representative immunofluorescence images of the CTraS-mediated grafts (upper panels) and sham-operated sample (lower panels) at 16 weeks after transplantation. Bars, 3 mm. R indicates the right side of the section. **(B)** Representative immunofluorescence images of grafted cells against SC-121, a human cytoplasmic marker, at 16 weeks after transplantation. Bars, 200 μm. **(C,D)** Immunostaining analysis of the graft at 16 weeks for mDA neuron-associated markers. Immunofluorescence images of indicated markers **(C)** and quantification of the marker-positive cells in HNA-positive cells **(D)**. Bars, 50 μm. **(E,F)** Immunofluorescence images of mDA neuron markers **(E)**, and quantification of marker-positive cells in HNA- and TH- double-positive cells **(F)**. Bars, 100 μm. **(G)** Immunofluorescence images of subtype-specific mDA neuron markers. Bars, 10 μm. **(H)** Immunofluorescence images of astrocyte markers. Bars, 100 μm. **(I)** Immunofluorescence images of VLMC markers. The COL1A1 antibody recognizes both human and rodent COL1A1, and hCOL1A1 antibody recognizes human-specific COL1A1. Arrows indicate human iPSC-derived COL1A1-positive cells, and arrowheads indicate mouse-derived COL1A1-positive cells. Bars, 100 μm. mDA, midbrain dopaminergic; 6-OHDA, 6-hydroxydopamine; PD, Parkinson’s disease; CTraS chemically transitional embryoid-body-like state; HNA, human nuclear antigen; TH, Tyrosine hydroxylase; VLMC, vascular leptomeningeal-like cell.

To evaluate the therapeutic potential for cell replacement therapy of mDA progenitors derived from iPSCs by our neurosphere-based differentiation method without FACS enrichment, we transplanted mDA progenitors into 6-OHDA-induced PD model mice. We injected 6-OHDA in the right striatum of CB17 severe combined immunodeficient (SCID) mice, and the motor symptoms of each mouse were evaluated by apomorphine-induced rotational behavior to confirm whether 6-OHDA disrupted striatal function and sufficiently induced PD symptoms. Three weeks after the 6-OHDA injection, we transplanted *in vitro* dissociated neurospheres on day 19 into the 6-OHDA-lesioned striatum (2 × 10^5^ cells in 2 μL), and injected saline in the sham group. Immunostaining with an SC-121 antibody, a human cytoplasmic marker, at 16 weeks after transplantation revealed that the transplanted cells survived and displayed neurite outgrowth in the striatum from the graft cells (Figures 2A,B).

To examine the composition of the graft cells, additional immunostaining was performed using mDA neuronal markers. The percentage of TH positive dopaminergic neurons was 14.0 ± 0.97% among human nuclear antigen (HNA, a human nuclear marker)-positive transplanted cells (Figures 2C,D). Among these HNA and TH double-positive neurons, 33.3 ± 5.68% were positive for the dopamine transporter (DAT), and 31.2 ± 5.23% were positive for aldehyde dehydrogenase 1A1 (ALDH1A1, an A9 mDA marker) (Figures 2E,F). These iPSC-derived dopaminergic neurons were also positive for mature mDA markers such as GIRK2 and Calbindin (Figure 2G). These results indicate that some transplanted iPSC-derived mDA progenitor cells have differentiated into mature dopaminergic neurons in the 4 months following transplantation. To evaluate the maturity of the graft cells, we immunostained brain sections with early mDA markers, FOXA2 and NURR1. The percentage of FOXA2+, early mDA progenitors, was 33.0 ± 2.7% and the percentage of NURR1+, postmitotic mDA progenitors, was 90.7 ± 1.3% in HNA-positive graft cells. Thus, the majority of the grafted cells were in the lineage of mDA neurons, and 14% of cells developed into mature mDA neurons. However, other cells remained at the progenitor stage (Figures 2C,D). The graft included 3.7 ± 0.49% of Ki67-positive proliferating cells and most of the Ki67-positive cells co-expressed FOXA2 (Ki67+/FOXA2+, 92.1 ± 2.06%), indicating that they were early mDA progenitors. The percentage of 5-HT+ serotonergic neurons was 4.7 ± 0.58%. These results indicate that transplanted mDA progenitors differentiated into ventral mDA neurons in the striatum of PD model mice with efficiencies similar to those reported earlier (Doi et al., 2014; Bye et al., 2015; Samata et al., 2016; Doi et al., 2020; Xu et al., 2022).

Recent single-cell analyses of transplanted ESC- and iPSC-derived ventral midbrain progenitor cells into rodent PD models have shown that the cells differentiate into neurons, astrocytes, and vascular leptomeningeal-like cells (VLMCs) (Tiklova et al., 2020; Xu et al., 2022). In the present study, we examined the expression of oligodendrocyte, astrocyte and VLMC markers by fluorescent immunohistochemistry to explore the possibility that our transplanted cells might differentiate into non-neuronal cells. A few grafted cells were positive for astrocyte markers, such as glial fibrillary acidic protein (GFAP) and S100β (Figure 2H). However, we found no evidence that grafted cells differentiate into oligodendrocytes and oligodendrocyte progenitor cells (OPCs) by staining against Olig2 and platelet-derived growth factor receptor (PDGFR) (Supplementary Figure S3). The presence of VLMC was assessed using a COL1A1 antibody that recognizes both human and rodent COL1A1, as well as a human-specific COL1A1 antibody (hCOL1A1). Within the grafts, we observed graft-derived human COL1A1-positive cells (Figure 2I, arrows) and infiltration of mouse-derived COL1A1-positive cells (Figure 2I, arrowheads). These results indicate that our transplanted mDA progenitors have similar differentiation potential to previously reported ESC- and iPSC-derived ventral midbrain progenitor cells.

### Grafted human iPSC-derived mDA neurons restored motor function in PD model mice

To evaluate the function of grafted mDA neurons, we performed a behavioral analysis of transplanted PD model mice using apomorphine-induced rotation every 4 weeks after transplantation. The 6-OHDA-lesioned PD mouse model rotates around to the left on being injected with apomorphine, due to the loss of dopaminergic neurons in the right striatum (Akerud et al., 2001; Grealish et al., 2010). A reduction in the number of rotations induced by apomorphine indicates the differentiation of dopaminergic neurons after the successful transplantation of dopaminergic progenitor cells, thus improving PD symptoms.

**Figure 3:**
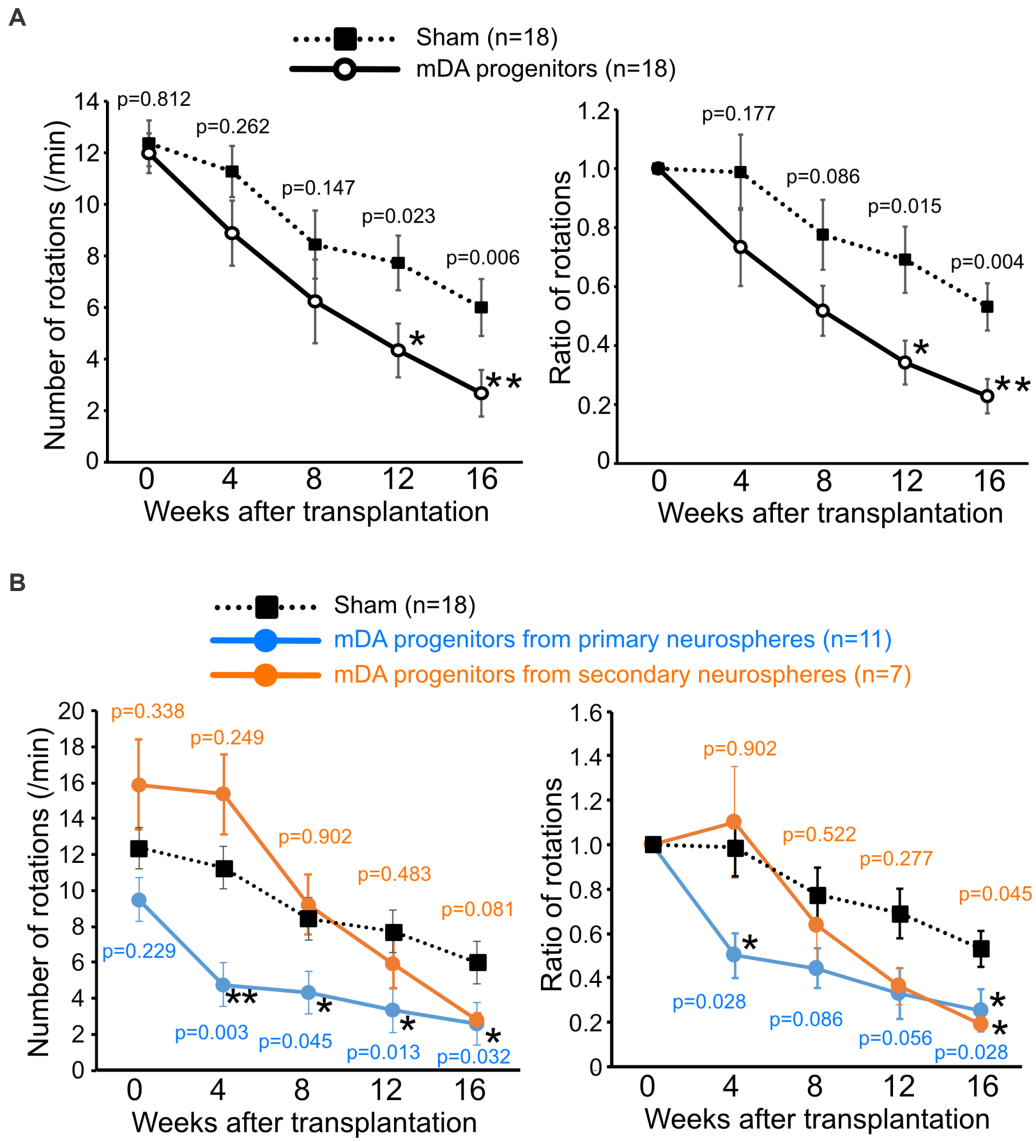
Grafted human iPSC-derived mDA neurons improve the motor functions of PD-model mice. The number of apomorphine-induced rotations (left) and the ratio to that of before the operation (right). **(A)** mDA progenitor transplantation: *n* = 18, sham: *n* = 18, mean ± SEM. The *p*-values were comparisons with the sham group at the same time-point using Student’s *t*-test. **(B)** Graph of results with separate the graft cells from primary neurospheres (*n* = 11) and secondary neurospheres (*n* = 7). The p-values were comparisons with the sham group at the same time-point using Steel’s multiple comparison test. *p < 0.05, **p < 0.01. iPSCs induced pluripotent stem cells; PD, Parkinson’s disease; mDA, midbrain dopaminergic.

A decrease in the number of rotations was observed in both cell-transplanted and sham-operated mice. However, 12 weeks after surgery, the number of rotations was significantly lower in the cell-transplanted mice than those in the sham-operated mice, presumably due to intrinsic factors which restored motor function (4.34 ± 0.90 times/min in transplanted mice versus 7.73 ± 1.10 times/min in sham mice). Furthermore, this significant improvement in motor function by the transplanted cells was confirmed until at least 16 weeks after transplantation (2.67 ± 0.59 times/min in transplanted mice versus 6.00 ± 0.95 times/min in sham mice; Figure 3A). Since we used mDA progenitors from primary neurospheres and secondary neurospheres for transplanted cells, the separate graphs for primary neurosphere- and secondary neurosphere-derived cells was shown in Figure 3B. Despite the reduced number of samples in each group, there is still a consistent general trend, although there has been a lower detection of statistically significant differences. Unexpectedly, pre-transplant symptoms tended to be stronger in mice implanted with secondary neurospheres and weaker in those implanted with primary neurospheres. However, there was no statistically significant difference between these three groups, and secondary neurospheres showed a trend toward improvement compared to the Sham group. Thus, we concluded that grafted mDA neurons functioned in PD model mice.

### The human iPSC-derived mDA progenitors by CTraS-based protocol may contribute to the safety of cell transplantation therapy *in vivo*

**Figure 4:**
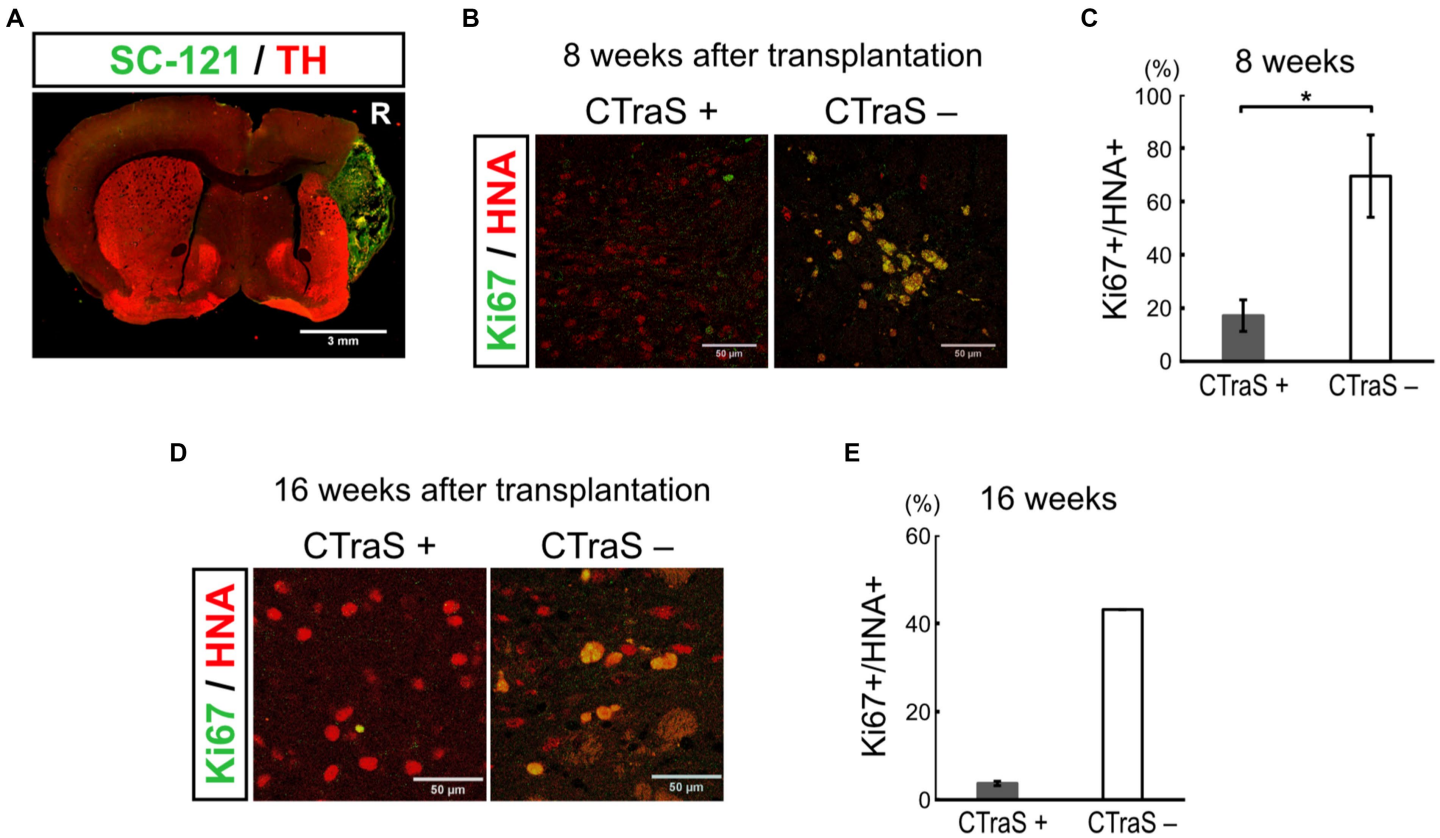
Grafted human iPSC-derived mDA neurons without CTraS method were likely to be tumors. **(A)** Immunofluorescence images of the aberrantly grown grafts, which was prepared without CTraS method at 8 weeks after transplantation. Bar, 3 mm. The right side of sections is indicated by R. **(B)** Immunofluorescence images of the graft at 8 weeks after transplantation. Bars, 50 μm. **(C)** Quantification of the ratio of Ki67-positive per human nuclear antigen (HNA)-positive cells. CTraS+: *n* = 3, CTraS-: *n* = 3, mean ± SEM. *p < 0.05. **(D)** Immunofluorescence images of the graft at 8 weeks after transplantation. **(E)** Quantification of the ratio of Ki67+/HNA+ cells. CTraS+: *n* = 13, CTraS-: *n* = 2, mean ± SEM. iPSCs, induced pluripotent stem cells; CTraS, chemically transitional embryoid-body-like state; HNA, human nuclear antigen.

We used the CTraS method to induce the mDA progenitor cells transplanted in this study. In this method, the treatment of undifferentiated iPSCs with three different inhibitors (SB, DM, and CHIR) accelerates the differentiation of subsequent cells (Fujimori et al., 2017). In this study, 18 PD model mice were transplanted with CTraS-mediated mDA progenitors and no tumor formation was observed. To evaluate the inhibitory effect of tumorigenesis using the CTraS method, we injected the same amounts of non-CTraS-mediated mDA progenitors, induced in the same protocol except iPSCs were not treated with the three inhibitors on days 0–5, into six PD model mice; three mice were analyzed at 8 weeks and the remaining three at 16 weeks after transplantation. At 8 weeks, one of three mice showed remarkable proliferation of transplanted cells which were considered to be the tumor (Figure 4A); a large number of the grafted cells in the other two mice were still Ki67-positive immature cells. The ratio of immature cells (Ki67+/HNA+, 69.4 ± 15.4%) was significantly higher than that of CTraS-mediated graft cells (17.2 ± 5.89%, Figures 4B,C). At 16 weeks, no tumor was found in the three mice; however, in one mouse, the transplanted cells were found outside the striatum. Although the sample size excluding mice with cells transplanted to different locations was too small for statistical analysis, many cells remained immature (43.1%) compared to those in CTraS-mediated graft cells (3.73 ± 0.49%, Figures 4D,E). These results indicate that this CTraS-based mDA neuron differentiation method potentially improves the safety of cell transplantation therapy in PD.

## Discussion

In the present study, to explore the therapeutic potential of human iPSC-derived CTraS-mediated dopaminergic progenitor cells for PD, we performed *in vivo* experiments using PD-model mice after *in vitro* evaluations. The transplanted progenitor cells showed high specificity for the ventral midbrain by expressing not only FOXA2, LMX1A, and NURR1, but also CORIN, ALCAM, LRTM1, CLSTN2 and PTPRO, which are potential cell surface markers to sort cells for transplantation therapy. In mice, postmitotic neuroblasts, which differentiate from radial glia (the mDA neuron progenitors) in the ventricular zone of the midbrain floor plate at E10.5, express NURR1, followed by TH, and as they mature they express mature mDA neuron markers such as GIRK2 and DAT (Zetterstrom et al., 1997; Arenas et al., 2015). The neuroblasts express NURR1 in addition to mDA neuron progenitor markers such as LMX1A, FOXA2, and EN1. The neurospheres are heterogeneous cell populations, and although they are capable of self-renewal, TH-positive cells were already observed on days 19 and 20 (Supplementary Figure S2), and TH expression increased as determined using qPCR (Supplementary Figure S1K). This suggests that the day 19 neurospheres correspond to approximately E10.5 to E12 in mice, and are a mixture of mDA neuron progenitors, NURR1-positive postmitotic neuroblasts, and TH-positive immature mDA neurons. Moreover, upon transferring to adhesion culture and neural differentiation medium to promote differentiation or by grafting, most cells appeared to be NURR1-positive postmitotic neuroblasts or mDA neurons. Our protocol provided a 79.2% FOXA2 positivity without sorting, which is comparable to previous reports with 75.5, 92.3, and 86.7% positivity in cells sorted with FOXA2, CORIN+ (Doi et al., 2014, 2020), LRTM1+ (Samata et al., 2016), respectively, while these values were unexamined in ALCAM+ (Bye et al., 2015) cells. Moreover, the efficiency of dopaminergic neuron induction *in vitro* was also similar to that obtained in previous reports using sphere culture and sorting; TH+/nucleus was 49.9% in this study compared to 42.0, 30–40, 46.3, and 43.1% using CORIN+ (Doi et al., 2014), LMX1A+ (Samata et al., 2016), CLSTN2+, and PTPRO (Xu et al., 2022), respectively. Therefore, our protocol could be useful in improving previous methods for preparing cells using floating neurosphere culture and sorting for transplantation therapy. In contrast to the floor plate method (Kriks et al., 2011), the efficiency of *in vitro* mDA neuron induction by Kriks et al. (2011) was approximately 80%, indicating that our method was deficient. However, the mDA neurons of grafted cells prepared using the floor plate method *in vivo* were 30% (Kriks et al., 2011), 19% (Kirkeby et al., 2012), 2% (Kim et al., 2021), and 9.2–9.3% (Piao et al., 2021), indicating that cells grafted using our method showed almost comparable differentiation efficiency.

In addition, *in vivo* behavioral experiments revealed improved motor symptoms in transplanted PD-model mice after 12 weeks compared with those in the sham group. The unilateral 6-OHDA lesion model is widely used in rats; however, it is difficult to reproduce the model in mice, at least in part, due to the smaller size of the mouse brain than that of rats (Grealish et al., 2010). Nevertheless, some researchers have generated models with stable motor impairments; Grealish et al. (2010) showed that the apomorphine-induced rotation behavior does not occur in 6-OHDA-lesioned mice with insufficient striatal destruction, requiring approximately 70% or more destruction (Grealish et al., 2010). Therefore, we used 6-OHDA lesion model mice, which exhibited at least seven rotations/min prior to transplantation but showed spontaneous recovery. Alvarez-Fischer et al. (2008) have shown in behavioral experiments that 6-OHDA injection into the mouse striatum causes a gradual recovery over 56 days (fixed speed rotarod and accelerating rotarod); however a decrease in the striatum DA levels and the number of substantia nigra TH+ cells persists for last at least 56 days (Alvarez-Fischer et al., 2008). Moerover spontaneous recovery from apomorphine-induced rotation movements in 6-OHDA lesioned mice has been reported in long-term observations (Bez et al., 2016). In our model mice, we estimate that approximately 70% striatal destruction was achieved; however, this may not be sufficient to produce stable motor deficits. Nevertheless, compared to the saline injected control group, the mDA progenitors transplant group showed clear improvement in motor symptoms and cell viability of transplanted cells, which indicates the effectiveness of cell transplant treatment in model mice. Additional behavioral experiments and experiments with other models, such as rats, would provide a more accurate evaluation.

In present study, the accelerated recovery of motor deficits observed suggested that iPSC-derived mDA progenitor cells derived using our method are differentiated into functional mDA neurons after transplantation, as reported in previous studies using rodent PD models (Doi et al., 2014; Han et al., 2015; Steinbeck et al., 2015; Samata et al., 2016; Doi et al., 2020). This interpretation was corroborated using pathological experiments, which revealed that the mDA progenitors differentiated into neurons, and 14% of the grafted neurons were positive for TH at 16 weeks post-transplantation. Although the comparison is not simple due to differences in the cell line and the species of the host, the TH+ ratio in this study was low compared to that in previous reports [18% of CORIN+ grafted cells at 16 weeks and 29.0% of LRTM+ grafted cells at 12 weeks in model rats (Doi et al., 2014, Samata et al., 2016)]. Moreover, a recent report revealed a TH positivity rate of 81.5 ± 3.5% among grafted cells in model rats sorted using CLSTN2 and 58.2 ± 3.5% among those sorted using PTPRO (Xu et al., 2022). However, all of these markers were expressed in mDA progenitor cells induced by our method (Supplementary Figures S1E,G,H,I), and it is expected that combining our method with sorting could further enhance the number of cells or improve purity.

The positive ratio of the proliferating cell marker Ki67 (3.7%) and the ratio of 5-HT-positive serotonergic neurons (4.7%) in this study were marginally higher than those reported in previous studies using cell sorting (0.06% Ki67+ and 1.2% 5-HT+ cells in CORIN+ grafts, and 1.8% Ki67+ and < 0.3% 5-HT+ cells in LRTM+ grafts) (Samata et al., 2016; Doi et al., 2020). However, 91% of the total surviving graft cells were positive for NURR1, a postmitotic mDA neuron progenitor marker. In our study, this percentage was considerably higher than those reported in studies using LRTM+ sorting (48%) (Samata et al., 2016). Most Ki67+ cells were also positive for the early mDA progenitor marker, FOXA2, in our study, which is similar to the results in LRTM+ cells (Samata et al., 2016). Thus, most grafted cells derived using our protocol without sorting differentiated in the lineage of mDA neurons to the same extent as previously reported cells derived using cell sorting; however, they were still immature and required time to develop into TH+ mature DA neurons. Another difference with previous reports is that we injected the cells without neurotrophic factors promoting neural survival and growth, such as brain-derived neurotrophic factor (BDNF), glial cell-derived neurotrophic factor (GDNF), and Y27632, a Rho-associated kinase inhibitor. Although we performed histological examinations at 16 weeks, improved results could be obtained if mDA progenitors were transplanted with neurotrophic factors and observed for a prolonged time.

The differentiation of transplanted ESC- and iPSC-derived ventral midbrain progenitor cells into non-neuronal cells has been reported based on single-cell analysis results (Tiklova et al., 2020; Xu et al., 2022). In these reports, grafted cells differentiated into astrocytes and VLMCs but not into OPCs or oligodendrocytes, which is consistent with our results. Moreover, a previous study using mesencephalic organoids has reported that dopaminergic neurons first differentiate from mesencephalic neural stem cells, followed by the appearance of VLMCs, astrocytes and OPCs (Fiorenzano et al., 2021). Considering that the appearance of non-neuronal cells was minimal in our *in vivo* experiments, it is possible that VLMC and astrocytes are only beginning to appear after 4 months of observation and that with longer-term observation, the appearance of non-neuronal cells may increase as dopaminergic neurons mature. The function of VLMCs in PD transplantation therapy is unknown; however, it has been suggested that they may be involved in the supportive effects of neurotrophic factor expression (Xu et al., 2022) and the formation of blood vessels to the graft by both host and graft cells (Tiklova et al., 2020). Further investigation of the therapeutic effect of non-neuronal cells using single-cell analysis is required.

As CTraS induction by treatment with the three chemicals promotes the differentiation of iPSCs into a three layer embryoid body-like state and shifts their differentiation into the final differentiated cells (Fujimori et al., 2017), this treatment could reduce undifferentiated cells that could be tumors. Consistent with this hypothesis, the grafted mDA progenitors using our protocol did not form tumors in all 18 mice, whereas non-CTraS-mediated mDA progenitors easily formed a tumor and there were significantly more mitotic immature cells in the other non-tumorigenic samples. While this result may indicate that our differentiation protocol potentially improves the safety of cell transplantation, further studies with a larger sample size are needed. Notably, tumorigenicity has not been reported as an issue in transplantation therapy studies using iPS cell-derived dopaminergic progenitor cells.

Based on the number of cells used in preclinical studies in monkeys (Kikuchi et al., 2017), approximately 5 × 10^6^ cells per patient are used in dopamine progenitor cell transplantation trials in patients with PD. At present, clinical-grade iPSCs, which are the source cells to induce the transplantation of dopamine progenitors, are developed from allogenic cell stocks that have been carefully tested for safety, mainly for economic reasons. If transplantation therapy moves to large-scale clinical trials and becomes a standard treatment option in the future, high cell production would have to be maintained. In such a case, sorting using FACS may become a rate-limiting step, as it may be difficult to prepare a large number of cells simultaneously. These disadvantages may be resolved if the cells produced by our induction method have the same safety and effectiveness as those reported earlier (Piao et al., 2021). However, to ensure the clinical applicability of our method, the treatment effects should be validated in larger animals, including primates, and further testing for the characterization of graft cells is required, as shown in the ongoing clinical trial (Kikuchi et al., 2017; Doi et al., 2020). Furthermore, rigorous and designed tumorigenesis studies are required, as well as some modifications to maintain good manufacturing practices, including clinical-grade feeder-free iPSCs, media and compounds. As these limitations are considered as future steps, the induction method presented in this study can help in the development of cell transplantation therapy for patients with PD.

In conclusion, mDA neuron progenitors for cell transplantation therapy for PD prepared by our protocol showed differentiation properties and therapeutic effects similar to the conventional method of cell sorting. Our protocol has the advantage of easily obtaining dopaminergic progenitors without the need for cell sorting. Further improvements in combination with known or new protocols may be applicable for actual clinical applications.

## Data availability statement

The raw data supporting the conclusions of this article will be made available by the authors, without undue reservation.

## Ethics statement

The animal study was reviewed and approved by the Ethics Review Committee for Animal Experimentation of Juntendo University School of Medicine.

## Author contributions

K-iI, GO, WA, and NH conceived and designed the experiments. RyN, K-iI, RiN, TJ, HK, and MN performed the experiments and analyzed the data. RyN, K-iI, GO, RiN, and WA wrote and revised the manuscript. All authors have reviewed and approved the manuscript.

## Funding

This work was funded by MEXT-Supported Programs for the Strategic Research Foundation at Private Universities (S1411007) and the Practical Research Project for Rare/Intractable Diseases (JP20ek0109429 to K-iI, GO, WA, and NH) from AMED, and a Grant-in-Aid for Scientific Research (JP20K07873 and JP23K06934 to K-iI, JP15K19498 and JP18K07509 to GO, JP20K07741 to RiN, and JP18H04043 to NH) from JSPS. This work was also supported in part by a Grant-in-Aid for Special Research in Subsidies for ordinary expenses of private schools from The Promotion and Mutual Aid Corporation for Private Schools of Japan.

## Conflict of interest

The authors declare that the research was conducted in the absence of any commercial or financial relationships that could be construed as a potential conflict of interest.

## Publisher’s note

All claims expressed in this article are solely those of the authors and do not necessarily represent those of their affiliated organizations, or those of the publisher, the editors and the reviewers. Any product that may be evaluated in this article, or claim that may be made by its manufacturer, is not guaranteed or endorsed by the publisher.

## References

[ref1] Akerud P. Canals J. M. Snyder E. Y. Arenas E. (2001). Neuroprotection through delivery of glial cell line-derived neurotrophic factor by neural stem cells in a mouse model of Parkinson's disease. J. Neurosci. 21, 8108–8118. doi: 10.1523/JNEUROSCI.21-20-08108.2001, PMID: 11588183 PMC6763865

[ref2] Alvarez-Fischer D. Henze C. Strenzke C. Westrich J. Ferger B. Hoglinger G. U.. (2008). Characterization of the striatal 6-Ohda model of Parkinson's disease in wild type and alpha-synuclein-deleted mice. Exp. Neurol. 210, 182–193. doi: 10.1016/j.expneurol.2007.10.012, PMID: 18053987

[ref3] Arenas E. Denham M. Villaescusa J. C. (2015). How to make a midbrain dopaminergic neuron. Development 142, 1918–1936. doi: 10.1242/dev.097394 26015536

[ref4] Barker R. A. Barrett J. Mason S. L. Björklund A. (2013). Fetal dopaminergic transplantation trials and the future of neural grafting in Parkinson's disease. Lancet Neurol. 12, 84–91. doi: 10.1016/S1474-4422(12)70295-8, PMID: 23237903

[ref5] Barker R. A. Drouin-Ouellet J. Parmar M. (2015). Cell-based therapies for Parkinson disease-past insights and future potential. Nat. Rev. Neurol. 11, 492–503. doi: 10.1038/nrneurol.2015.123 26240036

[ref6] Barker R. A. Kuan W. L. (2010). Graft-induced dyskinesias in Parkinson's disease: what is it all about? Cell Stem Cell 7, 148–149. doi: 10.1016/j.stem.2010.07.003 20682443

[ref7] Barker R. A. Parmar M. Studer L. Takahashi J. (2017). Human trials of stem cell-derived dopamine neurons for Parkinson's disease: dawn of a new era. Cell Stem Cell 21, 569–573. doi: 10.1016/j.stem.2017.09.014, PMID: 29100010

[ref8] Bateup H. S. Santini E. Shen W. Birnbaum S. Valjent E. Surmeier D. J.. (2010). Distinct subclasses of medium spiny neurons differentially regulate striatal motor behaviors. Proc. Natl. Acad. Sci. U. S. A. 107, 14845–14850. doi: 10.1073/pnas.1009874107, PMID: 20682746 PMC2930415

[ref9] Bez F. Francardo V. Cenci M. A. (2016). Dramatic differences in susceptibility to L-dopa-induced dyskinesia between mice that are aged before or after a nigrostriatal dopamine lesion. Neurobiol. Dis. 94, 213–225. doi: 10.1016/j.nbd.2016.06.005 27312773

[ref10] Brundin P. Nilsson O. G. Strecker R. E. Lindvall O. Astedt B. Bjorklund A. (1986). Behavioural effects of human fetal dopamine neurons grafted in a rat model of Parkinson's disease. Exp. Brain Res. 65, 235–240. PMID: 3542544 10.1007/BF00243848

[ref11] Bye C. R. Jonsson M. E. Bjorklund A. Parish C. L. Thompson L. H. (2015). Transcriptome analysis reveals transmembrane targets on transplantable midbrain dopamine progenitors. Proc. Natl. Acad. Sci. U. S. A. 112, E1946–E1955. doi: 10.1073/pnas.1501989112, PMID: 25775569 PMC4403171

[ref12] Chambers S. M. Fasano C. A. Papapetrou E. P. Tomishima M. Sadelain M. Studer L. (2009). Highly efficient neural conversion of human ES and iPS cells by dual inhibition of SMAD signaling. Nat. Biotechnol. 27, 275–280. doi: 10.1038/nbt.1529, PMID: 19252484 PMC2756723

[ref13] De Lau L. M. L. Breteler M. M. B. (2006). Epidemiology of Parkinson's disease. Lancet Neurol. 5, 525–535. doi: 10.1016/S1474-4422(06)70471-9 16713924

[ref14] Doi D. Magotani H. Kikuchi T. Ikeda M. Hiramatsu S. Yoshida K.. (2020). Pre-clinical study of induced pluripotent stem cell-derived dopaminergic progenitor cells for Parkinson's disease. Nat. Commun. 11:3369. doi: 10.1038/s41467-020-17165-w, PMID: 32632153 PMC7338530

[ref15] Doi D. Samata B. Katsukawa M. Kikuchi T. Morizane A. Ono Y.. (2014). Isolation of human induced pluripotent stem cell-derived dopaminergic progenitors by cell sorting for successful transplantation. Stem Cell Rep. 2, 337–350. doi: 10.1016/j.stemcr.2014.01.013, PMID: 24672756 PMC3964289

[ref16] Fasano C. A. Chambers S. M. Lee G. Tomishima M. J. Studer L. (2010). Efficient derivation of functional floor plate tissue from human embryonic stem cells. Cell Stem Cell 6, 336–347. doi: 10.1016/j.stem.2010.03.001 20362538 PMC4336800

[ref17] Fiorenzano A. Sozzi E. Birtele M. Kajtez J. Giacomoni J. Nilsson F.. (2021). Single-cell transcriptomics captures features of human midbrain development and dopamine neuron diversity in brain organoids. Nat. Commun. 12:7302. doi: 10.1038/s41467-021-27464-5, PMID: 34911939 PMC8674361

[ref18] Freed C. R. Greene P. E. Breeze R. E. Tsai W. Y. Dumouchel W. Kao R.. (2001). Transplantation of embryonic dopamine neurons for severe Parkinson's disease. N. Engl. J. Med. 344, 710–719. doi: 10.1056/NEJM200103083441002 11236774

[ref19] Fujimori K. Matsumoto T. Kisa F. Hattori N. Okano H. Akamatsu W. (2017). Escape from pluripotency via inhibition of TGF-Beta/BMP and activation of Wnt signaling accelerates differentiation and aging in hPSC progeny cells. Stem Cell Reports 9, 1675–1691. doi: 10.1016/j.stemcr.2017.09.024, PMID: 29107593 PMC5831048

[ref20] Grealish S. Mattsson B. Draxler P. Bjorklund A. (2010). Characterisation of behavioural and neurodegenerative changes induced by intranigral 6-hydroxydopamine lesions in a mouse model of Parkinson's disease. Eur. J. Neurosci. 31, 2266–2278. doi: 10.1111/j.1460-9568.2010.07265.x 20529122

[ref21] Han F. Wang W. Chen B. Chen C. Li S. Lu X.. (2015). Human induced pluripotent stem cell-derived neurons improve motor asymmetry in a 6-hydroxydopamine-induced rat model of Parkinson's disease. Cytotherapy 17, 665–679. doi: 10.1016/j.jcyt.2015.02.001, PMID: 25747741

[ref22] Ikeda A. Nishioka K. Meng H. Takanashi M. Hasegawa I. Inoshita T.. (2019). Mutations in CHCHD2 cause alpha-synuclein aggregation. Hum. Mol. Genet. 28, 3895–3911. doi: 10.1093/hmg/ddz241, PMID: 31600778

[ref23] Imaizumi K. Sone T. Ibata K. Fujimori K. Yuzaki M. Akamatsu W.. (2015). Controlling the regional identity of hPSC-derived neurons to uncover neuronal subtype specificity of neurological disease phenotypes. Stem Cell Reports 5, 1010–1022. doi: 10.1016/j.stemcr.2015.10.005, PMID: 26549851 PMC4682123

[ref24] Ishikawa K. I. Yamaguchi A. Okano H. Akamatsu W. (2018). Assessment of mitophagy in iPS cell-derived neurons. Methods Mol. Biol. 1759, 59–67. doi: 10.1007/7651_2017_10, PMID: 28324490

[ref25] Jakobs M. Fomenko A. Lozano A. M. Kiening K. L. (2019). Cellular, molecular, and clinical mechanisms of action of deep brain stimulation-a systematic review on established indications and outlook on future developments. EMBO Mol. Med. 11:e9575. doi: 10.15252/emmm.201809575, PMID: 30862663 PMC6460356

[ref26] Kalia L. V. Lang A. E. (2015). Parkinson's disease. Lancet 386, 896–912. doi: 10.1016/S0140-6736(14)61393-3 25904081

[ref27] Kefalopoulou Z. Politis M. Piccini P. Mencacci N. Bhatia K. Jahanshahi M.. (2014). Long-term clinical outcome of fetal cell transplantation for Parkinson disease: two case reports. JAMA Neurol. 71, 83–87. doi: 10.1001/jamaneurol.2013.4749, PMID: 24217017 PMC4235249

[ref28] Kikuchi T. Morizane A. Doi D. Magotani H. Onoe H. Hayashi T.. (2017). Human iPS cell-derived dopaminergic neurons function in a primate Parkinson's disease model. Nature 548, 592–596. doi: 10.1038/nature23664, PMID: 28858313

[ref29] Kim T. W. Piao J. Koo S. Y. Kriks S. Chung S. Y. Betel D.. (2021). Biphasic activation of WNT signaling facilitates the derivation of midbrain dopamine neurons from hESCs for translational use. Cell Stem Cell 28:E5. doi: 10.1016/j.stem.2021.01.005 PMC8006469 33545081

[ref30] Kirkeby A. Grealish S. Wolf D. A. Nelander J. Wood J. Lundblad M.. (2012). Generation of regionally specified neural progenitors and functional neurons from human embryonic stem cells under defined conditions. Cell Rep. 1, 703–714. doi: 10.1016/j.celrep.2012.04.009, PMID: 22813745

[ref31] Kriks S. Shim J. W. Piao J. Ganat Y. M. Wakeman D. R. Xie Z.. (2011). Dopamine neurons derived from human ES cells efficiently engraft in animal models of Parkinson's disease. Nature 480, 547–551. doi: 10.1038/nature10648, PMID: 22056989 PMC3245796

[ref32] Lindvall O. (2013). Developing dopaminergic cell therapy for Parkinson's disease-give up or move forward? Mov. Disord. 28, 268–273. doi: 10.1002/mds.25378 23401015

[ref33] Lindvall O. Rehncrona S. Brundin P. Gustavii B. Astedt B. Widner H.. (1989). Human fetal dopamine neurons grafted into the striatum in two patients with severe Parkinson's disease. A detailed account of methodology and a 6-month follow-up. Arch. Neurol. 46, 615–631. doi: 10.1001/archneur.1989.00520420033021, PMID: 2786405

[ref34] Lundblad M. Picconi B. Lindgren H. Cenci M. A. (2004). A model of L-dopa-induced dyskinesia in 6-hydroxydopamine lesioned mice: relation to motor and cellular parameters of nigrostriatal function. Neurobiol. Dis. 16, 110–123. doi: 10.1016/j.nbd.2004.01.007 15207268

[ref35] Matsumoto T. Fujimori K. Andoh-Noda T. Ando T. Kuzumaki N. Toyoshima M.. (2016). Functional neurons generated from T cell-derived induced pluripotent stem cells for neurological disease modeling. Stem Cell Reports 6, 422–435. doi: 10.1016/j.stemcr.2016.01.010, PMID: 26905201 PMC4788773

[ref36] Nolbrant S. Heuer A. Parmar M. Kirkeby A. (2017). Generation of high-purity human ventral midbrain dopaminergic progenitors for in vitro maturation and intracerebral transplantation. Nat. Protoc. 12, 1962–1979. doi: 10.1038/nprot.2017.078, PMID: 28858290

[ref37] Oji Y. Hatano T. Ueno S. I. Funayama M. Ishikawa K. I. Okuzumi A.. (2020). Variants in saposin D domain of prosaposin gene linked to Parkinson's disease. Brain 143, 1190–1205. doi: 10.1093/brain/awaa064, PMID: 32201884

[ref38] Olanow C. W. Goetz C. G. Kordower J. H. Stoessl A. J. Sossi V. Brin M. F.. (2003). A double-blind controlled trial of bilateral fetal nigral transplantation in Parkinson's disease. Ann. Neurol. 54, 403–414. doi: 10.1002/ana.10720, PMID: 12953276

[ref39] Parmar M. Torper O. Drouin-Ouellet J. (2019). Cell-based therapy for Parkinson's disease: a journey through decades toward the light side of the force. Eur. J. Neurosci. 49, 463–471. doi: 10.1111/ejn.14109, PMID: 30099795 PMC6519227

[ref40] Piao J. Zabierowski S. Dubose B. N. Hill E. J. Navare M. Claros N.. (2021). Preclinical efficacy and safety of a human embryonic stem cell-derived midbrain dopamine progenitor product, MSK-DA01. Cell Stem Cell 28:E7. doi: 10.1016/j.stem.2021.01.004 PMC7903922 33545080

[ref41] Piccini P. Brooks D. J. Bjorklund A. Gunn R. N. Grasby P. M. Rimoldi O.. (1999). Dopamine release from nigral transplants visualized in vivo in a Parkinson's patient. Nat. Neurosci. 2, 1137–1140. doi: 10.1038/16060, PMID: 10570493

[ref42] Politis M. Wu K. Loane C. Quinn N. P. Brooks D. J. Rehncrona S.. (2010). Serotonergic neurons mediate dyskinesia side effects in Parkinson's patients with neural transplants. Sci. Transl. Med. 2:38ra46. doi: 10.1126/scitranslmed.3000976 20592420

[ref43] Samata B. Doi D. Nishimura K. Kikuchi T. Watanabe A. Sakamoto Y.. (2016). Purification of functional human ES and iPSC-derived midbrain dopaminergic progenitors using LRTM1. Nat. Commun. 7:13097. doi: 10.1038/ncomms13097, PMID: 27739432 PMC5067526

[ref44] Schweitzer J. S. Song B. Herrington T. M. Park T. Y. Lee N. Ko S.. (2020). Personalized iPSC-derived dopamine progenitor cells for Parkinson's disease. N. Engl. J. Med. 382, 1926–1932. doi: 10.1056/NEJMoa1915872, PMID: 32402162 PMC7288982

[ref45] Shiba-Fukushima K. Ishikawa K. I. Inoshita T. Izawa N. Takanashi M. Sato S.. (2017). Evidence that phosphorylated ubiquitin signaling is involved in the etiology of Parkinson's disease. Hum. Mol. Genet. 26, 3172–3185. doi: 10.1093/hmg/ddx201, PMID: 28541509

[ref46] Steece-Collier K. Rademacher D. J. Soderstrom K. (2012). Anatomy of graft-induced dyskinesias: circuit remodeling in the parkinsonian striatum. Basal Ganglia 2, 15–30. doi: 10.1016/j.baga.2012.01.002, PMID: 22712056 PMC3375918

[ref47] Steinbeck J. A. Choi S. J. Mrejeru A. Ganat Y. Deisseroth K. Sulzer D.. (2015). Optogenetics enables functional analysis of human embryonic stem cell-derived grafts in a Parkinson's disease model. Nat. Biotechnol. 33, 204–209. doi: 10.1038/nbt.3124, PMID: 25580598 PMC5117952

[ref48] Suzuki S. Akamatsu W. Kisa F. Sone T. Ishikawa K. I. Kuzumaki N.. (2017). Efficient induction of dopaminergic neuron differentiation from induced pluripotent stem cells reveals impaired mitophagy in PARK2 neurons. Biochem. Biophys. Res. Commun. 483, 88–93. doi: 10.1016/j.bbrc.2016.12.188 28057485

[ref49] Takahashi J. (2020). iPS cell-based therapy for Parkinson's disease: a Kyoto trial. Regen Ther. 13, 18–22. doi: 10.1016/j.reth.2020.06.002, PMID: 33490319 PMC7794047

[ref50] Takahashi K. Tanabe K. Ohnuki M. Narita M. Ichisaka T. Tomoda K.. (2007). Induction of pluripotent stem cells from adult human fibroblasts by defined factors. Cells 131, 861–872. doi: 10.1016/j.cell.2007.11.019 18035408

[ref51] Thomson J. A. Itskovitz-Eldor J. Shapiro S. S. Waknitz M. A. Swiergiel J. J. Marshall V. S.. (1998). Embryonic stem cell lines derived from human blastocysts. Science 282, 1145–1147. doi: 10.1126/science.282.5391.1145 9804556

[ref52] Tiklova K. Nolbrant S. Fiorenzano A. Bjorklund A. K. Sharma Y. Heuer A.. (2020). Single cell transcriptomics identifies stem cell-derived graft composition in a model of Parkinson's disease. Nat. Commun. 11:2434. doi: 10.1038/s41467-020-16225-5, PMID: 32415072 PMC7229159

[ref53] Xu P. He H. Gao Q. Zhou Y. Wu Z. Zhang X.. (2022). Human midbrain dopaminergic neuronal differentiation markers predict cell therapy outcomes in a Parkinson's disease model. J. Clin. Invest. 132:e156768. doi: 10.1172/JCI156768, PMID: 35700056 PMC9282930

[ref54] Yamaguchi A. Ishikawa K. I. Inoshita T. Shiba-Fukushima K. Saiki S. Hatano T.. (2020). Identifying therapeutic agents for amelioration of mitochondrial clearance disorder in neurons of familial Parkinson disease. Stem Cell Reports 14, 1060–1075. doi: 10.1016/j.stemcr.2020.04.011, PMID: 32470327 PMC7355139

[ref55] Zetterstrom R. H. Solomin L. Jansson L. Hoffer B. J. Olson L. Perlmann T. (1997). Dopamine neuron agenesis in Nurr1-deficient mice. Science 276, 248–250. doi: 10.1126/science.276.5310.248, PMID: 9092472

[ref56] Zuo F. Xiong F. Wang X. Li X. Wang R. Ge W.. (2017). Intrastriatal transplantation of human neural stem cells restores the impaired subventricular zone in parkinsonian mice. Stem Cells 35, 1519–1531. doi: 10.1002/stem.2616, PMID: 28328168

