# Supplementary material for: A defined method for differentiating human iPSCs into midbrain dopaminergic progenitors that safely restore motor deficits in Parkinson’s disease
Source: Front Neurosci. 2023 Jul 12;17:1202027. doi: 10.3389/fnins.2023.1202027 (PMC10368972; doi:10.3389/fnins.2023.1202027)
Supplement: Supplementary file 1 [file Data_Sheet_1.docx]

Supplementary Material

A defined method for differentiating human iPSCs into midbrain dopaminergic progenitors that safely restore motor deficits in Parkinson’s disease

Ryota Nakamura, Risa Nonaka, Genko Oyama, Takayuki Jo, Hikaru Kamo, Maierdanjiang Nuermaimaiti, Wado Akamatsu, Kei-ichi Ishikawa*, Nobutaka Hattori*

*** Correspondence:** Kei-ichi Ishikawa: [kishikaw@juntendo.ac.jp](mailto:kishikaw@juntendo.ac.jp); and Nobutaka Hattori: nhattori@juntendo.ac.jp

# Supplementary Figures and Tables

## Supplementary Figures


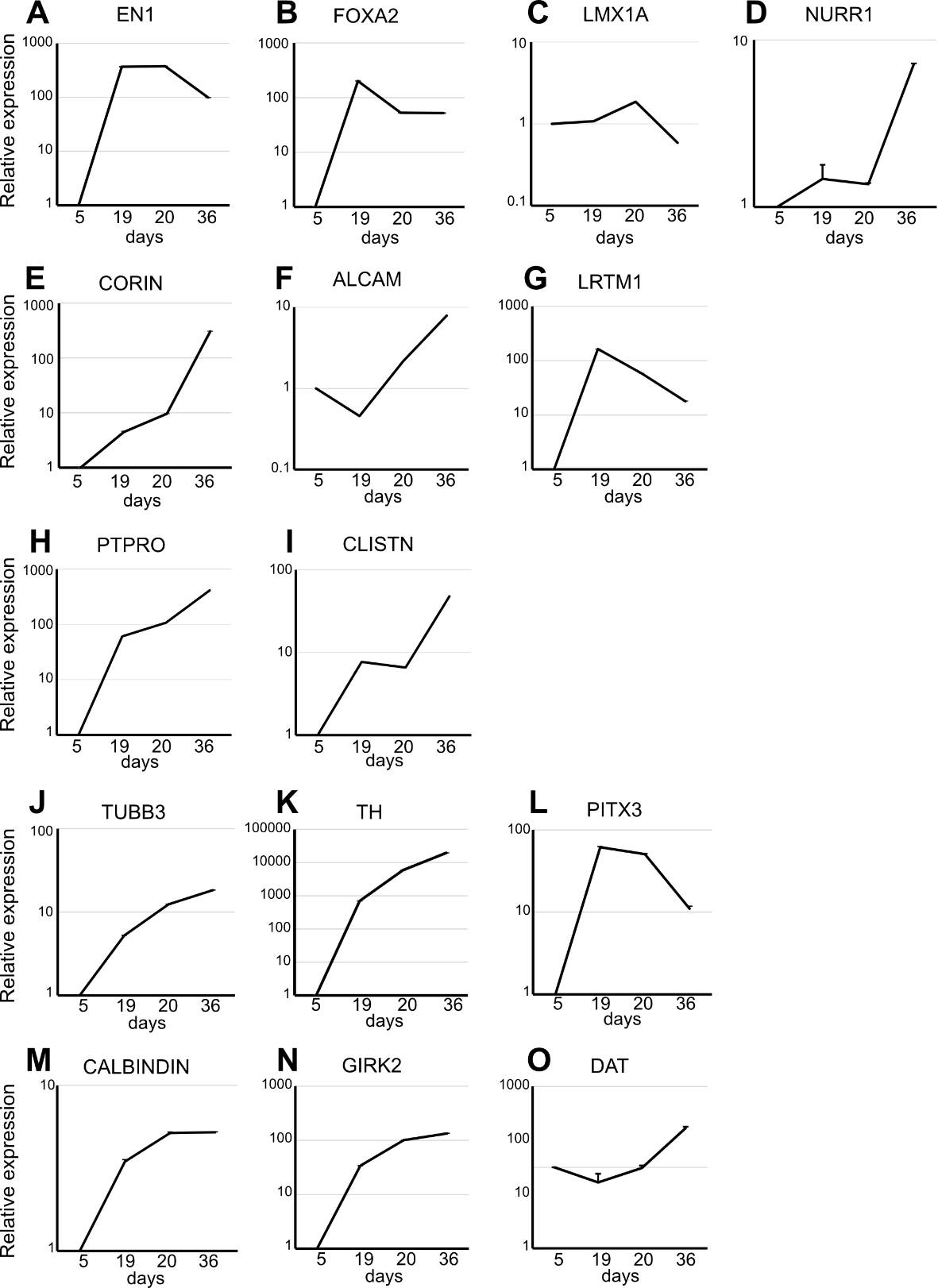


**Supplementary Figure 1.** **Gene expression analysis during midbrain dopaminergic (mDA) neuron induction from induced pluripotent stem (iPS) cells.**

Quantitative real time-PCR analysis of markers in CTraS (day 5), mDA progenitors of neurospheres (day 19) and of attached cultures (day 20), and mDA neurons (day 36). Values were normalized to *ACTB* and were analysed using the comparative (ΔΔCt) method. n =3–4.


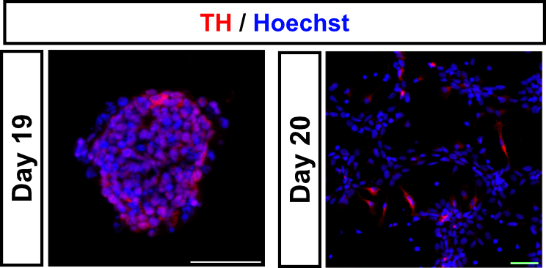


**Supplementary Figure S2. Immunostaining analysis of mDA progenitors.**

Immunofluorescence images of mDA progenitors on days 19 and 20 for TH. Scale bar, 50 μm.

**
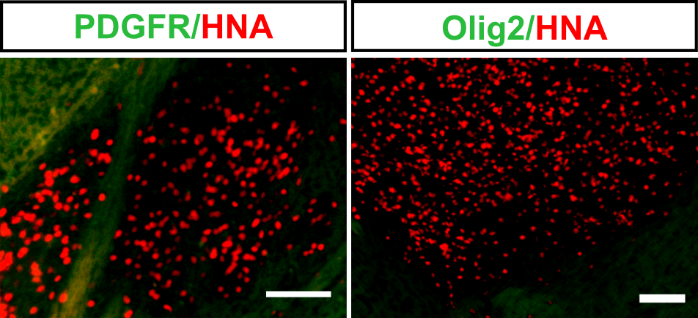
**

**Figure S3. Immunostaining analysis of grafted cells.**

Representative immunofluorescence images of grafted cells against PDGFR, an oligodendrocyte progenitor cell marker, and Olig2, an oligodendrocyte marker, at 16 weeks after transplantation. Scale bar, 100 μm

## Supplementary Tables

**Supplementary Table S1. Primers used for Quantitative RT-PCR.**

| Targets |  | 5′-sequence-3′ |
| --- | --- | --- |
| FOXG1 | Forward | 5′-AGAAGAACGGCAAGTACGAGA-3′ |
|  | Reverse | 5′-TGTTGAGGGACAGATTGTGGC-3′ |
| SIX3 | Forward | 5′-ACCGGCCTCACTCCCACACA-3′ |
|  | Reverse | 5′-CGCTCGGTCCAATGGCCTGG-3′ |
| EN1 | Forward | 5′-TGGGTGTACTGCACACGTTATTC-3′ |
|  | Reverse | 5′-GGAACTCCGCCTTGAGTCTCT-3′ |
| HOXB4 | Forward | 5′-CGTGAGCACGGTAAACCCCAA-3′ |
|  | Reverse | 5′-ATTCCTTCTCCAGCTCCAAGACCT-3′ |
| ACTB | Forward | 5′-TGAAGTGTGACGTGGACATC-3′ |
|  | Reverse | 5′-GGAGGAGCAATGATCTTGAT-3′ |
| FOXA2 | Forward | 5′-CCGTTCTCCATCAACAACCT-3′ |
|  | Reverse | 5′-GGGGTAGTGCATCACCTGTT-3′ |
| LMX1A | Forward | 5′-GTCCCAGAACCATCCTGACC-3′ |
|  | Reverse | 5′-GGAACCACACCTGAACCACA-3′ |
| NR4A2 | Forward | 5′-CGAAACCGAAGAGCCCACAGGA-3′ |
| (NURR1) | Reverse | 5′-GGTCATAGCCGGGTTGGAGTCG-3′ |
| CORIN | Forward | 5′-CACAGCCAGGGTCTGGTGGAATGCAG-3′ |
|  | Reverse | 5′-GAGAGCTACCACCACATGAATCAAGG-3′ |
| ALCAM | Forward | 5′-CCTTGTTGCTGGTGTCGTCTACT-3′ |
|  | Reverse | 5′-ATTACCGAGGTCCTTGTTTACATGT-3′ |
| LRTM1 | Forward | 5′-ATTGCCACTTGCTCGGTCTT-3′ |
|  | Reverse | 5′-TCCTTTCCCTTCCAGGTGTCT-3′ |
| PTPRO | Forward | 5′-ACAAGTTGGCTCCAGTCAGA-3′ |
|  | Reverse | 5′-GACGGCTATGAACGTAGGGA-3′ |
| CLSTN2 | Forward | 5′-CGTGCTCTCCAGAAAGTCTCC-3′ |
|  | Reverse | 5′-CTTCCCCAAAGCACTGGACTT-3′ |
| TUBB3 | Forward | 5′-ATTTCATCTTTGGTCAGAGTGGGGC-3′ |
|  | Reverse | 5′-TGCAGGCAGTCGCAGTTTTCAC-3′ |
| TH | Forward | 5′-TCATCACCTGGTCACCAAGTT-3′ |
|  | Reverse | 5′-GGTCGCCGTGCCTGTACT-3′ |
| PITX3 | Forward | 5′-CCTGCTTGCCTTCCAGACTGC-3′ |
|  | Reverse | 5′-ACTGGTCCCTATTCCTGGCCTTAGT-3′ |
| CALB1 | Forward | 5′-CCCTCAAGAACAAACAGAAACGA-3′ |
|  | Reverse | 5′-TGCTTCCATTTACACGCTAAAGG-3′ |
| KCNJ6 | Forward | 5′-CTGGAAATTGTGGTCATC-3′ |
| (GIRK2) | Reverse | 5′-GGTCTCATAGGTCTCATG-3′ |
| SLC6A3 | Forward | 5′-CCAGCTACAACAAGTTCACCAA-3′ |
| (DAT) | Reverse | 5′-AGAAGCTCGTCAGGGAGTTG-3' |

**Supplementary Table S2. List of antibodies used for immunofluorescence.**

| Targets | Host species | Label | Dilution | Source | Catalog number | RRID |
| --- | --- | --- | --- | --- | --- | --- |
| HNA | mouse | - | 1:400 | Merck Millipore | MAB1281 | AB_94090 |
| STEM121 | mouse | - | 1:500 | StemCells, Inc. | AB-121-U-050 | AB_2632385 |
| TH | sheep | - | 1:500 | Merck Millipore | AB1542 | AB_90755 |
| TH | rabbit | - | 1:500 | Merck Millipore | AB152 | AB_390204 |
| βⅢ tubulin | mouse | - | 1:1000 | Sigma-Aldrich | T8660 | AB_477590 |
| βⅢ tubulin | rabbit | - | 1;1000 | Covance | 845501 | AB_2566588 |
| FOXA2 | goat | - | 1:500, 1:200 | R&D Systems | AF2400 | AB_2294104 |
| NURR1 | rabbit | - | 1:100 | Abcam | ab227260 | - |
| NURR1 | mouse | - | 1:500 | Preseus proteomics | PP-N1404-00 | AB_2251476 |
| GIRK2 | rabbit | - | 1:200 | Alomone Labs | APC-006 | AB_2040115 |
| PITX3 | rabbit | - | 1:500 | Merck Millipore | AB5722 | AB_91997 |
| Ki67 | rabbit | - | 1:1000 | Novocastra | NCL-Ki67p | AB_442102 |
| 5-HT | rabbit | - | 1:10000 | Immunostar | 20080 | AB_572263 |
| LMX1A | rabbit | - | 1:250 | Abcam | ab31006 | AB_10601106 |
| LMX-1 | rabbit | - | 1:500 | Merck Millipore | AB10533 | AB_10805970 |
| CORIN | rat | - | 1:100 | R&D Systems | MAB2209 | AB_2082224 |
| LRTM1 | rabbit | - | 1:50 | Abcam | ab121409 | AB_11130788 |
| LRTM1 | rabbit | - | 1:100 | R&D Systems | MAB10046 | - |
| ALCAM | goat | - | 1:100 | R&D systems | AF1172 | AB_354644 |
| DAT | rabbit | - | 1:100 | Abcam | ab184451 |  |
| ALDH1A1 | rabbit | - | 1:1000 | Sigma | HPA002123 |  |
| S100β | rabbit | - | 1:100 | Abcam | ab52642 |  |
| GFAP | chicken | - | 1:200 | Abcam | ab5541 |  |
| COL1A1 | rabbit | - | 1:200 | Abcam | ab34710 |  |
| hCOL1A1 | sheep | - | 1:200 | R&D systems | AF6220 |  |
| PDGFR | rabbit | - | 1:300 | Santa Cruz | sc-338 |  |
| Olig2 | rabbit | - | 1:500 | R&D systems | AF2418 |  |
| CALBINDIN | mouse | - | 1:50 | Santa Cruz | sc-365360 | AB_10841576 |
| CALBINDIN | rabbit | - | 1:100 | Abcam | ab11426 | AB_298031 |
| CALBINDIN | mouse | - | 1:1000 | Swant Swiss | #300 | AB_10000347 |
| Anti-mouse IgG | goat | Alexa 488 | 1:500 | Invitrogen | A11001 | AB_2534069 |
| Anti-rabbit IgG | goat | Alexa 488 | 1:500 | Invitrogen | A11008 | AB_143165 |
| Anti-rabbit IgG | goat | Alexa 555 | 1:500 | Invitrogen | A21429 | AB_2535850 |
| Anti-rabbit IgG | donkey | Alexa 488 | 1:200 | Invitrogen | A21206 | AB_2535792 |
| Anti-rabbit IgG | donkey | Alexa 647 | 1:200 | Invitrogen | A31573 | AB_2536183 |
| Anti-rat IgG | donkey | Alexa 488 | 1:200 | Invitrogen | A21208 | AB_2535794 |
| Anti-sheep IgG | donkey | Alexa 555 | 1:200 | Invitrogen | A21436 | AB_2535857 |
| Anti-goat IgG | donkey | Alexa 555 | 1:200 | Invitrogen | A21432 | AB_2535853 |
| Anti-goat IgG | donkey | Alexa 568 | 1:200 | Invitrogen | A11057 | AB_2534104 |
| Anti-goat IgG | donkey | Alexa 647 | 1:200 | Invitrogen | A21447 | AB_2535864 |
